# Epstein-Barr Virus-Positive Lymphoproliferative Disorder in an HIV Patient Successfully Treated With Rituximab

**DOI:** 10.7759/cureus.92816

**Published:** 2025-09-21

**Authors:** Alen G Karapetians, Tejasvi Ayyagari, Wilbur Montana

**Affiliations:** 1 Internal Medicine and Hematology/Oncology, University of California, Los Angeles (UCLA) Kern Medical Center, Bakersfield, USA; 2 Oncology, University of California, Los Angeles (UCLA) Kern Medical Center, Bakersfield, USA

**Keywords:** ebv in hiv positive, ebv positive, ebv-positive b-cell lymphoproliferative disease, ebv-positive lymphoproliferative disorder, ebv-positive mucocutaneous ulcer, ebv ptld, epstein-barr virus (ebv), rituximab monotherapy, rituximab therapy

## Abstract

Epstein-Barr virus (EBV)-associated lymphoproliferative disorders (LPDs) represent a heterogeneous group of conditions that occur most often in immunocompromised individuals, including those with human immunodeficiency virus (HIV) infection, organ transplantation, autoimmune disease, and immune senescence. We report a case of a 55-year-old female with previously undiagnosed HIV, who presented with progressive throat pain, dysphagia, weight loss, and recurrent antibiotic-refractory tonsillitis, initially raising concern for squamous cell carcinoma. Imaging with contrast-enhanced CT of the neck revealed asymmetric oropharyngeal swelling with a rim-enhancing lesion, and biopsy demonstrated EBV-positive polymorphic LPD with clonal B-cell proliferation, confirmed by immunoglobulin heavy-chain gene rearrangement studies and Epstein-Barr virus-encoded RNA (EBER) in situ hybridization (EBER-ISH). Despite initiation of antiretroviral therapy and *Pneumocystis jirovecii* prophylaxis, the patient’s oropharyngeal symptoms and cervical lymphadenopathy progressed, accompanied by EBV viremia. Rituximab was introduced, resulting in rapid symptomatic relief, regression of lymphadenopathy, clearance of EBV viremia, and continued HIV viral suppression. This case highlights the diagnostic complexity of HIV-associated EBV-positive LPDs, which may mimic infection or malignancy and require biopsy with immunophenotyping, EBV detection, and clonality studies for accurate diagnosis. Furthermore, it underscores that while immune restoration with antiretroviral therapy is essential, it may not be sufficient to control EBV-driven lymphoproliferation, and targeted therapy with rituximab can be critical for achieving remission. Broader recognition of HIV-associated EBV-positive LPD within evolving classification frameworks is needed, along with standardized treatment strategies and prospective outcome studies to guide management of this rare but clinically significant disorder.

## Introduction

Epstein-Barr virus (EBV)-associated lymphoproliferative disorders (LPDs) encompass a heterogeneous group of conditions characterized by abnormal proliferation of EBV-infected B cells, most often arising in immunocompromised individuals. These include patients with human immunodeficiency virus (HIV), organ transplantation, chemotherapy-induced immunosuppression, autoimmune disease, and immune senescence [1,2]. EBV has a well-established role in driving lymphoproliferative disease, ranging from benign mucocutaneous ulcers to aggressive lymphomas.

In HIV-infected individuals, EBV-positive LPDs are relatively uncommon compared to other immunodeficiency settings, with only limited case series reported. They may present with polymorphic histology, multifocal lesions, and symptoms mimicking infection or malignancy, making diagnosis particularly challenging [3,4]. Updated classification frameworks, including the 5th edition of the World Health Organization Classification of Haematolymphoid Tumours (WHO-HAEM5) and the International Consensus Classification (ICC), now recognize HIV-associated EBV-positive LPD as a distinct subgroup within the EBV-driven spectrum [2,5]. Here, we describe the case of a 55-year-old female with previously undiagnosed HIV who developed an EBV-positive polymorphic LPD initially mistaken for an oropharyngeal abscess, underscoring the diagnostic and therapeutic complexities in this population.

This article was previously presented as an iPoster abstract at the 6th Annual Southern San Joaquin Valley Regional Research Forum on May 22, 2025.

## Case presentation

A 55-year-old female with uncontrolled diabetes presented with a two-month history of progressive left-sided throat pain, foul purulent discharge, dysphagia, odynophagia, intermittent fever, chills, and weight loss. She had been evaluated multiple times for recurrent tonsillitis and was refractory to several antibiotic regimens. Neck CT revealed asymmetric oropharyngeal swelling with a rim-enhancing hypodense lesion and stable cervical lymphadenopathy. The rim-enhancing appearance initially raised concern for a deep neck space abscess, particularly given the patient’s history of recurrent tonsillitis and purulent discharge. However, the absence of a well-formed fluid collection and the persistence of symptoms despite multiple antibiotic courses also raised the possibility of a necrotic tonsillar squamous cell carcinoma or other malignant process. These overlapping radiologic features contributed to the diagnostic uncertainty and prompted tissue biopsy for definitive diagnosis.

She was admitted for biopsy to exclude malignancy, with tonsillar squamous cell carcinoma as the leading differential diagnosis. Intraoperatively, a friable mass with exudate and granulation tissue was found in the left tonsillar fossa with uvular erosion. Pending results of the biopsy, the patient's HIV screening returned positive, with RNA quantification of 546,000 copies/mL and a CD4 count of 204 cells/µL. Antiretroviral therapy (ART) with bictegravir/emtricitabine/tenofovir (Biktarvy) and *Pneumocystis jirovecii* prophylaxis with atovaquone (due to sulfa allergy) were initiated.

**Figure 1:**
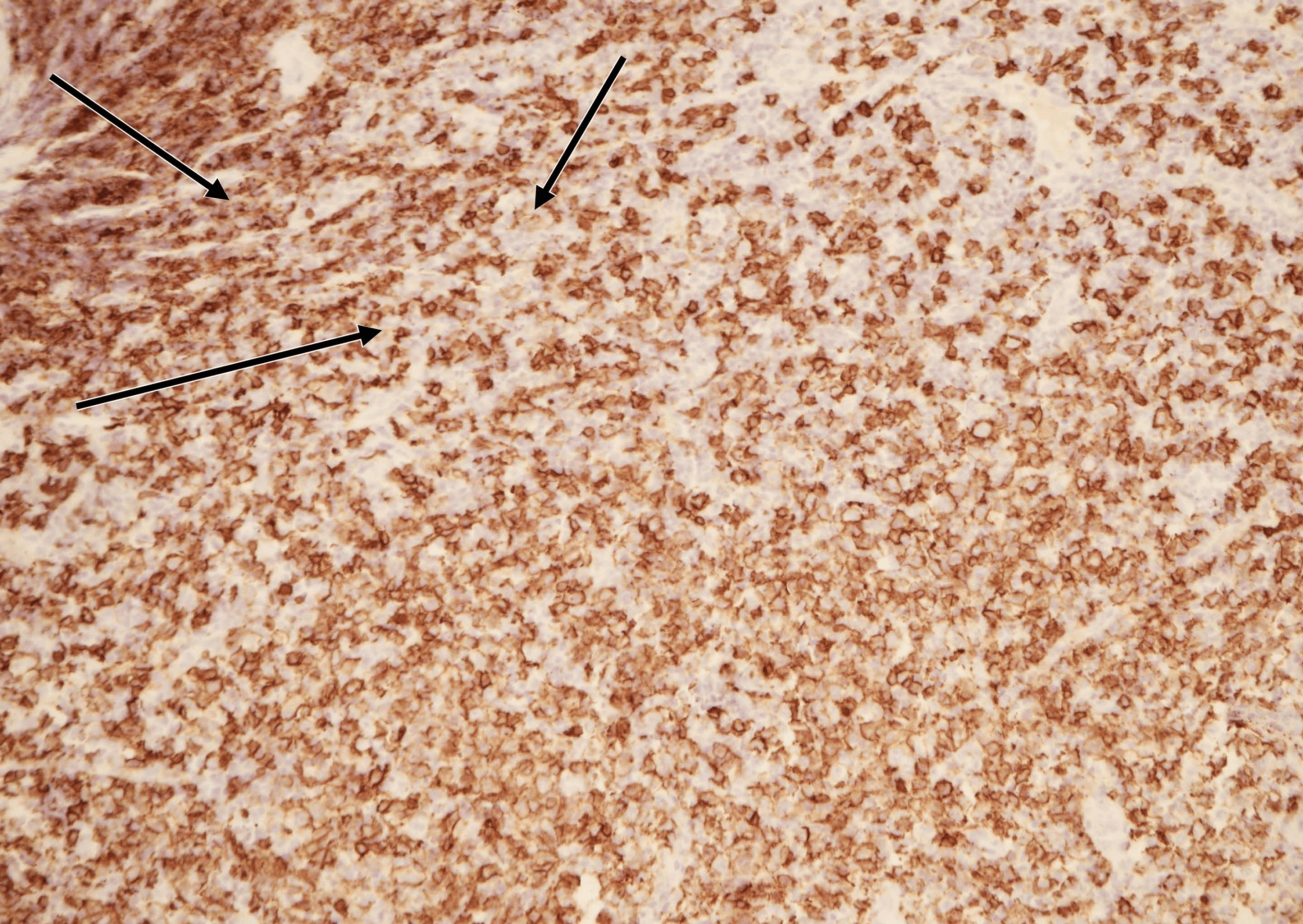
CD20 immunohistochemistry of tonsillar biopsy. Immunohistochemical staining demonstrates diffuse membranous expression of CD20 in atypical B cells (arrows), confirming a B-cell lineage process consistent with lymphoproliferative disorder.

**Figure 2:**
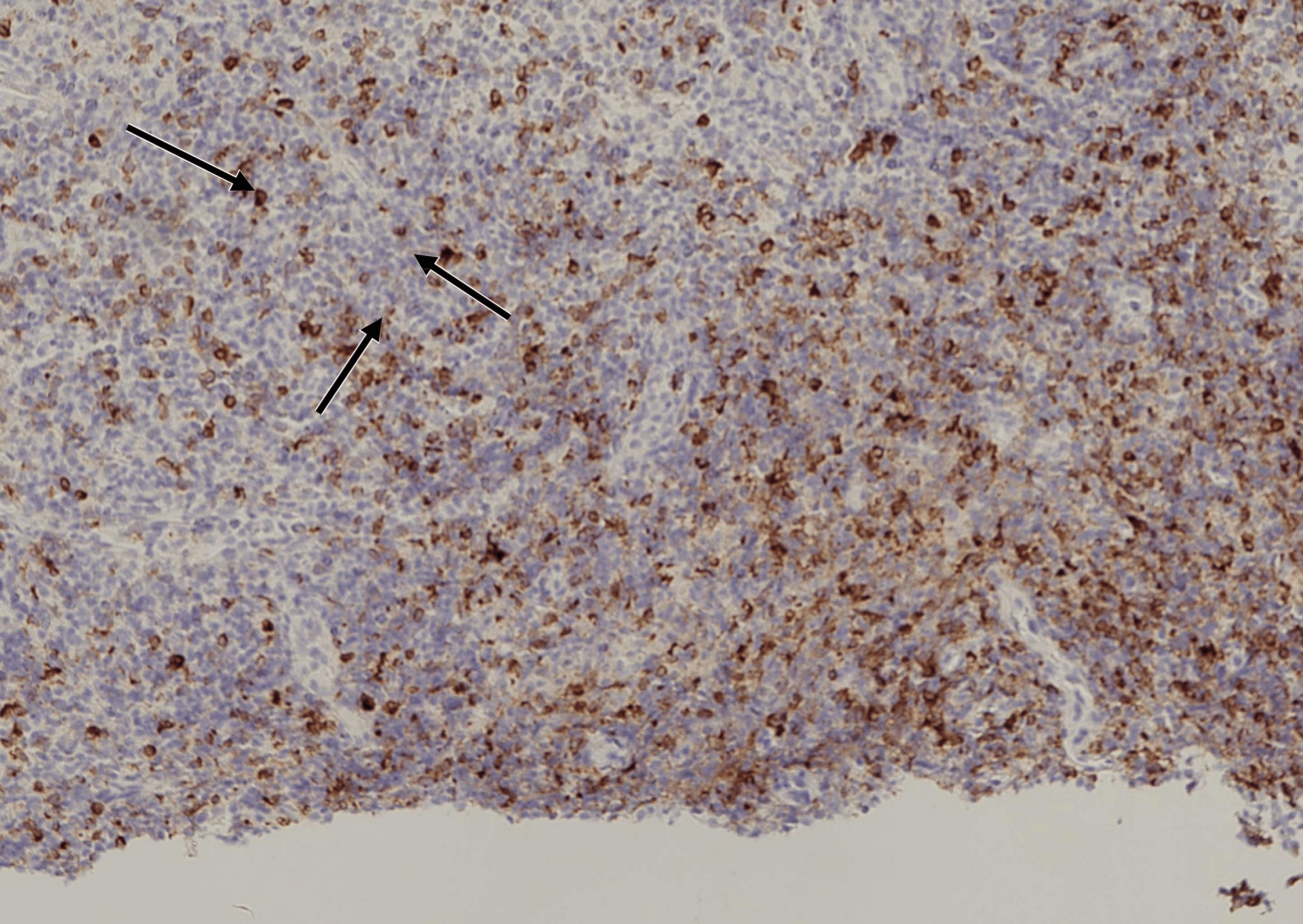
CD79a immunohistochemistry of tonsillar biopsy. A subset of atypical lymphoid cells shows weak cytoplasmic CD79a expression (arrows), supporting B-cell origin while highlighting the immunophenotypic variability often seen in EBV-positive lymphoproliferative disorders.

**Figure 3:**
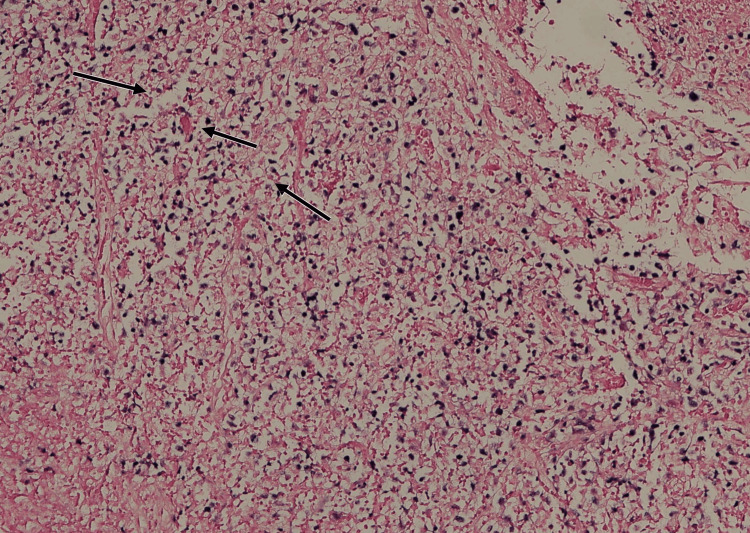
Epstein–Barr virus (EBV) in situ hybridization of tonsillar biopsy. Epstein-Barr virus-encoded RNA (EBER) in situ hybridization reveals nuclear positivity (arrows) in numerous atypical B cells, providing direct confirmation of EBV involvement in the lymphoproliferative process.

**Figure 4:**
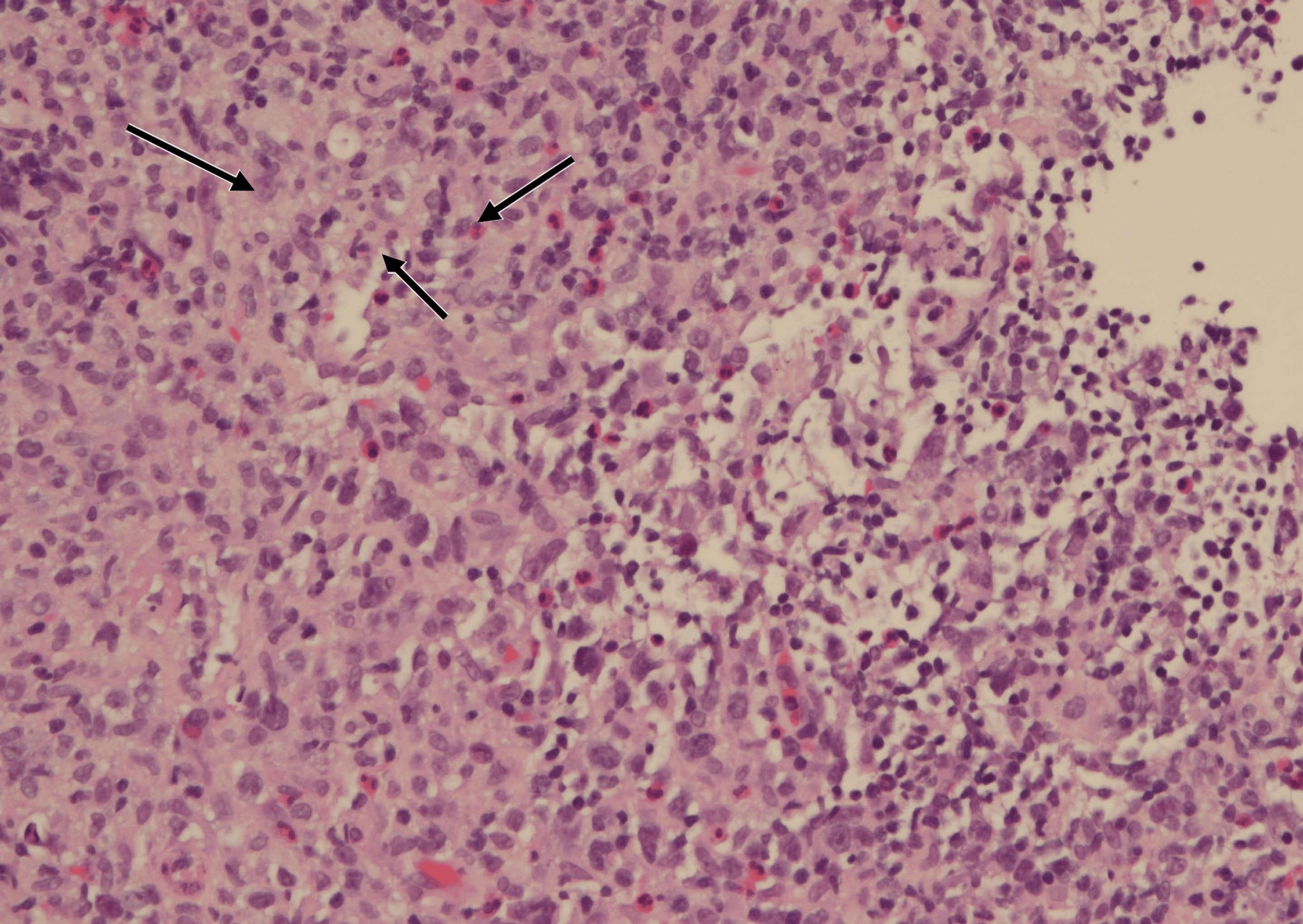
Hematoxylin and eosin (H&E) stain of tonsillar biopsy (20× magnification). Histology demonstrates a polymorphic infiltrate of atypical lymphoid cells with admixed histiocytes and eosinophils (arrows), a classic morphologic pattern supporting Epstein-Barr virus-positive polymorphic lymphoproliferative disorder in the setting of HIV immunosuppression.

Histopathology confirmed EBV-positive polymorphic LPD. The biopsy demonstrated atypical B-cell proliferation with diffuse membranous CD20 positivity (Figure 1) and weak cytoplasmic CD79a staining in a subset of atypical cells (Figure 2). Epstein-Barr virus-encoded RNA (EBER) in situ hybridization (EBER-ISH) showed nuclear positivity in many of the atypical lymphoid cells (Figure 3). Hematoxylin and eosin (H&E) staining revealed a polymorphic infiltrate composed of small to large atypical lymphoid cells in a background of histiocytes and eosinophils (Figure 4). Clonality was confirmed using immunoglobulin heavy-chain gene rearrangement studies.

The patient re-presented shortly after discharge with worsening odynophagia, inability to tolerate oral medications, and persistent dysphagia. Repeat imaging showed bilateral deep cervical lymphadenopathy and pharyngeal mucosal edema. EBV polymerase chain reaction was positive with a viral load of 11,069 copies/mL, while HIV RNA had decreased to 66 copies/mL on therapy. Despite immune restoration with ART, symptoms persisted. Rituximab was initiated (five weekly infusions), resulting in rapid clinical improvement after the first dose. By treatment completion, the patient’s throat pain and dysphagia had resolved, cervical lymphadenopathy regressed, EBV viral load became undetectable, and HIV RNA declined to <20 copies/mL (Table 1).

**Table 1:** Clinical course, laboratory values, and treatment timeline. EBV: Epstein-Barr virus; ART: antiretroviral therapy; LPD: lymphoproliferative disorder; EBER: Epstein-Barr virus-encoded RNA; PCP: Pneumocystis pneumonia; PTLD: post-transplant lymphoproliferative disorder.

Time point	Key events	HIV RNA (copies/mL)	CD4 count (cells/µL)	EBV DNA (copies/mL)	Treatment/intervention	Clinical response
Presentation	Progressive throat pain, dysphagia, recurrent tonsillitis; CT showed a rim-enhancing lesion	546,000	204	Not obtained	Supportive care; admitted for biopsy	Persistent symptoms
Hospitalization	Tonsillar mass biopsy: EBV-positive polymorphic LPD confirmed (CD20+, EBER+)	546,000	204	Not obtained	ART initiated (Biktarvy), PCP prophylaxis with atovaquone	Discharged, but symptoms persisted
2 weeks post ART	Worsening odynophagia and cervical lymphadenopathy	66	Not repeated	11,069	Continued ART; no additional therapy yet	No improvement
Rituximab initiation	Started weekly rituximab infusions (x5)	—	—	11,069	Rituximab 375 mg/m² IV weekly × 5 (standard PTLD dosing)	Rapid improvement after the first dose
End of rituximab course	Completion of 5 infusions	<20	Not reported	Undetectable	Continued ART	Resolution of throat pain, dysphagia, and lymphadenopathy

## Discussion

EBV-positive LPDs are well-recognized complications of immunodeficiency and share striking similarities across diverse immunosuppressive settings. HIV-associated EBV-positive LPDs closely resemble post-transplant lymphoproliferative disorders (PTLD) both histologically and clinically, as both are driven by EBV-induced clonal B-cell proliferation under impaired immune surveillance [1,4]. Consequently, therapeutic strategies for HIV-associated EBV-positive LPD are largely extrapolated from PTLD management guidelines.

While immune restoration through ART is critical in HIV, ART alone may not be sufficient for controlling EBV-driven disease. In our case, despite virologic suppression of HIV, EBV-related lymphoproliferation persisted until rituximab was introduced. Rituximab, a monoclonal antibody against CD20-positive B cells, interrupts EBV-driven clonal expansion and has demonstrated effectiveness in both PTLD and HIV-associated EBV-positive LPD through a limited number of case reports and small series [3,6]. This case reinforces rituximab’s role as an effective adjunct when ART alone is inadequate; however, further studies are needed as current supporting data are limited.

EBV DNA quantification has been validated as a surrogate marker of tumor burden and treatment response in PTLD and is increasingly applied in HIV-associated EBV-positive LPDs [7]. In our patient, the presence of systemic EBV viremia was a key feature distinguishing polymorphic EBV-positive LPD from EBV-associated mucocutaneous ulcer (EBVMCU), which shares overlapping histology but is usually localized and EBV-viremia negative [8].

Current classification schemes, including the WHO-HAEM5 and ICC, have expanded to incorporate EBV-driven LPDs across diverse immune deficiency contexts such as PTLD, autoimmune disease, inborn errors of immunity, chemotherapy-related LPD, HIV infection, and immune senescence [2,5]. This evolving taxonomy underscores the need for tailored management strategies in each setting.

## Conclusions

This case highlights the diagnostic challenges of EBV-associated LPD in HIV, where the initial presentation may closely mimic common infections or malignancies of the oropharynx. Definitive diagnosis requires biopsy with immunophenotyping, EBV detection, and clonality studies, supported by viral load monitoring. While immune restoration with ART remains the foundation of management, EBV-related lymphoproliferation may persist despite adequate HIV control. In our patient, rituximab was necessary to achieve rapid clinical remission, regression of adenopathy, and clearance of EBV viremia.

More broadly, this case underscores the need to incorporate HIV-associated EBV-positive LPD into evolving classification frameworks and to develop standardized management strategies distinct from post-transplant settings. Future studies should clarify the role of EBV DNA monitoring, define when to escalate from ART alone to targeted immunotherapy, and establish outcome data specific to HIV-related disease. Prospective research and multicenter registries will be essential in shaping guidelines for this rare but clinically significant entity.
